# Clinical characteristics and outcomes of patients with Corona Virus Disease 2019 (COVID-19) at Mercy Health Hospitals, Toledo, Ohio

**DOI:** 10.1371/journal.pone.0250400

**Published:** 2021-04-22

**Authors:** Muhammad Shayan Khan, Ratika Dogra, Leela K. V. Miriyala, F. N. U. Salman, Rizwan Ishtiaq, Dilnoor K. Patti, Aakash Kumar, Gaurav Sandho, Karim Jacob, Kritika Luthra, Ravina Sharma, Rekha Ravikumar, Dharmakaruna Edara, Shanti Pittampalli, Divya Sood, Vinod Khatri, Vijay Mahajan, Salil Avasthi, Arlette Auoad, Srinivas Katragadda

**Affiliations:** 1 Internal Medicine Residency, Mercy St Vincent Medical Centre, Toledo, Ohio, United States of America; 2 Pulmonary and Critical Care, Mercy St Vincent Medical Centre, Toledo, Ohio, United States of America; 3 Infectious Diseases, Mercy St Vincent Medical Centre, Toledo, Ohio, United States of America; Dasman Diabetes Institute, KUWAIT

## Abstract

**Importance:**

The ongoing pandemic of the novel Corona Virus Disease 2019 (COVID-19) is an unprecedented challenge to global health, never experienced before.

**Objective:**

This study aims to describe the clinical characteristics and outcomes of patients with COVID-19 admitted to Mercy Hospitals.

**Design and methods:**

Retrospective, observational cohort study designed to include every COVID-19 subject aged 18 years or older admitted to Mercy Saint (St) Vincent, Mercy St Charles, and Mercy St Anne’s hospital in Toledo, Ohio from January 1, 2020 through June 15^th^, 2020. Primary Outcome Measure was mortality in the emergency department or as an in-patient.

**Results:**

470 subjects including 224 males and 246 females met the inclusion criteria for the study. Subjects with the following characteristics had higher odds (OR) of death: Older age [OR 8.3 (95% CI 1.1–63.1, p = 0.04)] for subjects age 70 or more compared to subjects age 18–29); Hypertension [OR 3.6 (95% CI 1.6–7.8, p = 0.001)]; Diabetes [OR 3.1 (95% CI 1.7–5.6, p<0.001)]; COPD [OR 3.4 (95% CI 1.8–6.3, p<0.001)] and CKD stage 2 or greater [OR 2.5 (95% CI 1.3–4.9, p = 0.006)]. Combining all age groups, subjects with hypertension had significantly greater odds of the following adverse outcomes: requiring hospital admission (OR 2.2, 95% CI 1.4–3.4, p<0.001); needing respiratory support in 24 hours (OR 2.5, 95% CI: 1.7–3.7, p<0.001); ICU admission (OR 2.7, 95% CI 1.7–4.4, p<0.001); and death (OR 3.6, 95% CI 1.6–7.8, p = 0.001). Hypertension was not associated with needing vent in 24 hours (p = 0.07).

**Conclusion:**

Age and hypertension were associated with significant comorbidity and mortality in Covid-19 Positive patients. Furthermore, people who were older than 70, and had hypertension, diabetes, COPD, or CKD had higher odds of dying from the disease as compared to patients who hadn’t. Subjects with hypertension also had significantly greater odds of other adverse outcomes.

## Background and significance

The ongoing pandemic of the novel Coronavirus Disease 2019 (COVID-19) is an unprecedented challenge to global health, never experienced before. This disease is caused by a virus, severe acute respiratory syndrome coronavirus 2 (SARS CoV-2), which belongs to a family of coronaviruses that also includes the SARS and Middle East Respiratory Syndrome (MERS) coronaviruses [1,2]. This disease has spread exponentially ever since the first case was reported in December 2019 in Wuhan China and was named the 2019 Novel Coronavirus and declared a pandemic officially by the World Health Organization (WHO) on 12th March 2020 [3]. As of Jan 5^th^, 2021, more than 83 million cases have been confirmed with more than 346,000 deaths worldwide [4].

Coronaviruses belong to a family of viruses that have been known to cause various problems ranging from mild fever and breathing difficulties to a full-blown lung infection and pneumonia **[5]**. SARS-CoV-2 belongs to the β-coronaviridae cluster, making it the 3rd known zoonotic disease linked to the coronavirus family (after SARS-CoV-1 and MERS) [6]. It may present in a variety of ways, ranging from fever (88%) to cough (68%), vomiting (5%), and diarrhea (3.8%) [7]. Many theories have been proposed regarding its pathophysiology and disease process. One theory suggests that it preferentially binds to the angiotensin-converting enzyme 2 (ACE-2) in the body, thus increasing its expression and resulting in damage to alveolar cells. These damaged alveolar cells in turn lead to a series of inflammatory bodily responses which cause the pattern of acute respiratory distress syndrome and even death [8].

Given the rapid spread of this virus with major effects on global health, there is a lot of interest in the scientific community. One important area of concern is the high mortality among study participants who require mechanical support with some studies showing mortality as high as 61% in patients with a known outcome [9,10]. The following study aims to describe patient characteristics and overall outcomes of COVID-19 positive subjects admitted to Mercy Health Hospitals in the Toledo Region. The main objectives were as following:

1. To describe the epidemiological characteristics and co-morbidities of adult cases of COVID-19 admitted (ED or in-patient) to Mercy Hospitals (St. Vincent, St. Charles, St. Anne’s) between 1/1/2020 and 6/15/2020, overall and by age group.
2. To explore epidemiological characteristics and co-morbidities univariately and multivariately associated with in-hospital mortality among COVID-19 patients.
3. To describe outcomes of COVID patients (i.e., required hospital admission, required ICU admission, needed respiratory support or vent in 24 hours, died in hospital), overall, by age group, and separately for cases with and without hypertension co-morbidity.
4. To explore factors univariately and multivariately associated with admission to intensive care unit, need for ventilator or oxygen in first 24 hours, and total hospital length of stay

## Methods

### Study design

This retrospective cohort study was approved by the Institutional Review Board (IRB) for Mercy Health System, Toledo with a waiver of consent and a waiver of Protected Health Information.

### Subjects and inclusion criteria

Patients were included if they had a confirmed diagnosis of COVID-19 by the real-time polymerase chain reaction (PCR) and criteria devised by the WHO interim guidance [11]. Thus, every COVID-19 subject admitted to Mercy Health Hospitals who were discharged alive or died while in the emergency department or in-patient from St. Vincent, St. Charles, or St. Anne’s medical facility from January 1, 2020 through June 15th, 2020 were included in our study. Outcome data was obtained after June 15th, 2020 only on those patients who were admitted on or before June 15th, 2020 but remained inpatient afterward until discharged or deceased.

### Exclusion criteria

Subjects less than 18 years of age.

### Primary and secondary outcome measures

The primary outcome measure was mortality in the emergency department or as an in-patient.

The secondary outcome measures were ICU stay, need for a ventilator or respiratory support in the first 24 hours, total hospital length of stay in days.

### Data collection

Data was collected using a retrospective chart review using the electronic medical records of all patients. Epidemiological, clinical, laboratory, and radiological characteristics, along with treatment and outcome data were obtained. Outcome information was collected on these patients until discharge or death in the hospital. Information recorded for purpose of the study included De-Identified Code (Patient number), Hospital, Date of birth, Date of admission, Gender, Race, Length of Stay (LOS), Age Category, Body Mass Index (BMI), Weight, height, Co-morbidities, D-Dimer on admission, highest D-Dimer levels, Respiratory Support Need within 24 hours, Ventilator settings, Compliance (Static and Dynamic), Proned (Yes/No), Thrombophilia panel, Treatment, ICU admission (Yes/No), number of ICU days, Complications and Disposition.

### Statistical methods

Continuous variables were expressed as median and interquartile range (IQR). The frequency of categorical variables was reported as number and percentage. Factors associated with in-hospital mortality were explored univariately with logistic regression. The odds ratio (OR) for death was reported with 95% confidence interval and Wald Chi-square p-value. Confidence intervals that did not contain 1 were considered significant without adjustment for multiple comparisons. Data was analyzed using SAS V9.4.

## Main results

A total of 471 subjects met the inclusion criteria for the study and were included in the research dataset. One was missing age and therefore 470 subjects including 224 males (47.7%) and 246 females (52.3%) were studied.

More than half of the patients, 237 (50.4%) were seen at Mercy St Vincent Hospital which serves as the main referral center in the Toledo region. This was followed by St Anne’s and then St Charles Hospital. Around Three quarters (74%) of the patients were older than 50 years of age, this trend was reciprocated across all three hospitals. No significant sex preponderance was present. Almost half of the patients were white [238 (48.1%)], this was followed by African Americans [169 (38.2%)], and then Latinos [42 (9.5%)]. The most common co-morbidity was hypertension (62.8%), followed by diabetes (40.4%) and then COPD (18.5%). The majority of co-morbidities were in the older age group, with 48.1% of the total patient sample having 2 or more co-morbidities. Out of these, 43.4% were >50 years old and 46.5% were >70 years old. Although the mean D-Dimer on admission increased from being 0.50 mcg/ml in the 18–29 age group to 2.26 mcg/ml in the >70 subset, this was not associated with a statistically increased risk of admission. 76.4% of the patients seen in the ER were admitted whereas the rest were discharged. Out of those admitted, the majority (68.4%) were discharged home, 20.7% people to rehab/nursing facilities, and 10.9% people passed away. Out of the diseased subset, more than 94% of the people were aged > 50 years with 56.9% patients > 70 years old. Demographic and clinical characteristics of Covid-19 subjects are described in Table 1.

**Table 1:** Demographic and clinical characteristics of COVID subjects, total and by age group.

	Subjects by age				
	All n (% of all)	18–29 n (% of row)	30–49 n (% of row)	50–69 n (% of row)	70 or more n (% of row)
No. (%)	470	38 (8.1)	84 (17.9)	190 (40.4)	158 (33.6)
Hospital					
St. Vincent	237 (50.4)	22 (9.3)	42 (17.7)	102 (43.0)	71 (30.0)
St. Charles	89 (18.9)	9 (10.1)	23 (25.8)	31 (34.8)	26 (29.2)
St. Annes	144 (30.6)	7 (4.9)	19 (13.2)	57 (39.6)	61 (42.4)
Sex					
Male	224 (47.7)	16 (7.1)	43 (19.2)	92 (41.1)	73 (32.6)
Female	246 (52.3)	22 (8.9)	41 (16.7)	98 (39.8)	85 (34.6)
Race					
White	213 (48.1)	14 (6.6)	23 (10.8)	82 (38.5)	94 (44.1)
African American	169 (38.2)	15 (8.9)	37 (21.9)	80 (47.3)	37 (21.9)
Hispanic-Latino	42 (9.5)	1 (2.4)	17 (40.9)	13 (31.0)	11 (26.2)
Multiracial	7 (1.6)	4 (57.1)	1 (14.3)	2 (28.6)	0 (0)
Other	12 (2.7)	0 (0)	3 (25.0)	5 (41.7)	4 (33.3)
BMI					
0–18	12 (2.7)	1 (8.3)	1 (8.3)	2 (16.7)	8 (66.7)
18–25	86 (19.2)	9 (10.5)	9 (10.5)	29 (33.7)	39 (45.4)
25–30	115 (25.6)	8 (7.0)	20 (17.4)	44 (38.3)	43 (37.4)
30 or more	236 (52.6)	19 (8.1)	53 (22.5)	110 (46.6)	54 (22.9)
Comorbidities					
Hypertension	295 (62.8)	4 (1.4)	37 (12.5)	134 (45.4)	120 (40.7)
Diabetes	190 (40.4)	0 (0)	32 (16.8)	80 (42.1)	78 (41.1)
CAD	77 (16.4)	0 (0)	3 (3.9)	27 (35.1)	47 (61.0)
COPD	87 (18.5)	2 (2.3)	6 (6.9)	40 (46.0)	39 (44.8)
CKD 2 or higher	74 (15.7)	0 (0)	3 (4.1)	26 (35.1)	45 (60.8)
PAD	25 (5.3)	0 (0)	1 (4.0)	11 (44.0)	13 (52.0)
Other	156 (33.2)	4 (2.6)	23 (14.7)	67 (43.0)	62 (39.7)
2 or more comorbidities	226 (48.1)	1 (0.4)	22 (9.7)	98 (43.4)	105 (46.5)
D-dimer on admit median (IQR)	1.04 (0.58, 2.57)	0.50 (0.29, 1.01)	0.76 (0.49, 1.04)	1.04 (0.57, 1.96)	2.26 (0.98, 4.00)
Admission status					
Admitted (LOS≥1)	359 (76.4)	27 (7.5)	71 (19.8)	148 (41.2)	113 (31.5)
Not admitted (LOS = 0)	111 (23.6)	11 (9.9)	13 (11.7)	42 (37.8)	45 (40.5)
Disposition					
Discharged home	321 (68.4)	35 (10.9)	72 (22.4)	135 (42.1)	79 (24.6)
Discharged to rehab	97 (20.7)	2 (2.1)	9 (9.3)	36 (37.1)	50 (51.6)
Deceased	51 (10.9)	1 (2.0)	2 (3.9)	19 (37.3)	29 (56.9)

2 or more comorbidities is defined as having 2 or more of HTN, DM, CAD, COPD, CKD, PAD. LOS is hospital length of stay.

Missing data were as follows: 27 subjects missing race, 21 BMI, 260 d-dimer, 1 disposition. Percentages may sum to more or less than 100 due to rounding.

Subjects with the following characteristics had higher odds of death: Older age [OR8.3 (95% CI 1.1–63.1, p = 0.04)] for subjects age 70 or more compared to subjects age 18–29); Hypertension [OR 3.6 (95% CI 1.6–7.8, p = 0.001)]; Diabetes [OR 3.1 (95% CI 1.7–5.6, p<0.001)]; COPD [OR 3.4 (95% CI 1.8–6.3, p<0.001)], and CKD stage 2 or greater [OR 2.5 (95% CI 1.3–4.9, p = 0.006)] (Table 2). Out of the 51 diseased patients, 84.3% had hypertension, this was followed by diabetes (64.7%), COPD (39.2%) and CKD stage 2 or greater (29.4%). 76.5% of diseased patients had 2 or more co-morbidities. Race was not associated with increased odds of death. As compared to the Caucasian race, the OR for African American population for mortality was 0.7 (95% CI 0.4–1.4, p = 0.37) and all other races was 1.3 (95% CI 0.6–3.0, p = 0.53). Similarly, no significant association of increasing BMI with mortality could be demonstrated.

**Table 2:** Demographic factors associated with mortality among COVID-19 subjects admitted to the hospital or emergency room.

	Died n (% of deaths)	Discharged Alive n (% of alive)	Odds Ratio for Death [95% CI]	Wald Chi-square P-value
No. (% of all)	51(10.9)	419 (89.1)		
Age				overall p = 0.002
18–29	1 (2.0)	37 (8.9)	reference	reference
30–49	2 (3.9)	81 (19.4)	0.9 [0.1, 10.4]	0.94
50–69	19 (37.3)	171 (40.9)	4.1 [0.5, 31.7]	0.17
70 or more	29 (56.9)	129 (30.9)	8.3 [1.1, 63.1]	0.04
Sex				
Male	31 (60.8)	194 (46.3)	reference	
Female	20 (39.2)	225 (53.7)	0.6 (0.3, 1.0)	0.053
Race				overall p = 0.42
White	25 (51.0)	188 (47.7)	reference	reference
African American	15 (30.6)	154 (39.1)	0.7 [0.4, 1.4]	0.37
All Other Races	9 (18.4)	52 (13.2)	1.3 [0.6, 3.0]	0.53
BMI				overall p = 0.92
0–18	1 (2.0)	11 (2.8)	reference	reference
18–25	10 (20.4)	76 (19.0)	1.4 [0.2, 12.4]	0.74
25–30	14 (28.6)	100 (25.0)	1.5 [0.2, 12.8]	0.69
30 or more	24 (49.0)	213 (53.3)	1.2 [0.2, 10.0]	0.84
Comorbidities				
Hypertension	43 (84.3)	252 (60.1)	3.6 [1.6, 7.8]	0.001
Diabetes	33 (64.7)	157 (37.5)	3.1 [1.7, 5.6]	<0.001
CAD	12 (23.5)	65 (15.5)	1.7 [0.8, 3.4]	0.15
COPD	20 (39.2)	67 (16.0)	3.4 [1.8, 6.3]	<0.001
CKD 2 or higher	15 (29.4)	59 (14.1)	2.5 [1.3, 4.9]	0.006
PAD	5 (9.8)	20 (4.8)	2.2 [0.8, 6.1]	0.14
2 or more comorbidities	39 (76.5)	187 (44.6)		
D-dimer on admit median (IQR)	1.53 (0.59, 3.73)	1.02 (0.57, 2.22)	1.03 [1.0, 1.1]	0.11

IQR is interquartile range. CI is confidence interval, 95% Wald confidence interval. OR for d-dimer is the increase in odds per 1 unit increase in d-dimer.

After stratifying patients by presence or absence of hypertension, 36% of non-hypertensive patients required respiratory support within 24 hours, this was significantly lower than 59% of hypertensive patients requiring respiratory support in the same time period. Similar trends were observed regarding mortality and ICU admission. Combining all age groups, subjects with hypertension had significantly greater odds of the following adverse outcomes (Table 3): requiring hospital admission (OR 2.2, 95% CI 1.4–3.4, p<0.001); needing respiratory support in 24 hours (OR 2.5, 95% CI: 1.7–3.7, p<0.001); ICU admission (OR 2.7, 95% CI 1.7–4.4, p<0.001); and death (OR 3.6, 95% CI 1.6–7.8, p = 0.001). Hypertension was not associated with needing vent in 24 hours (p = 0.07).

**Table 3:** Outcomes of COVID subjects admitted to the hospital or emergency room, total and by age group.

	Subjects by age				
	All n (% of all)	18–29 n (% of row)	30–49 n (% of row)	50–69 n (% of row)	70 or more n (% of row)
No.(%)	470	38 (8.1)	84 (17.9)	190 (40.4)	158 (33.6)
**Overall**					
Required admission	359 (76.4)	27 (7.5)	71 (19.8	148 (41.2)	113 (31.5)
Needed respiratory support in 24 hours	237 (50.4)	9 (3.8)	39 (16.5)	105 (44.3)	84 (35.4)
Needed vent in 24 hours	69 (14.7)	6 (8.7)	8 (11.6)	33 (47.8)	22 (31.9)
Admitted to ICU	116 (24.7)	6 (5.2)	16 (13.8)	54 (46.6)	40 (34.5)
Died in hospital	51 (10.9)	1 (2.0)	2 (3.9)	19 (37.3)	29 (56.9)
**Subjects with hypertension**					
No. Subjects	295	4 (1.4)	37 (12.5)	134 (45.4)	120 (40.7)
Required admission	241 (81.7)	3 (1.2)	33 (13.7)	108 (44.8	97 (40.3)
Needed respiratory support in 24 hours	174 (59.0)	2 (1.2)	19 (10.9)	80 (46.0)	73 (42.0)
Needed vent in 24 hours	50 (16.9)	1 (2.0)	3 (6.0)	26 (52.0)	20 (40.0)
Admitted to ICU	91 (30.8)	2 (2.2)	7 (7.7)	45 (49.5)	37 (40.7)
Died in hospital	43 (14.6)	0 (0)	1 (2.3)	16 (37.2)	26 (60.5)
**Subjects without hypertension**					
No. Subjects	175	34 (19.4)	47 (26.9)	56 (32.0)	38 (21.7)
Required admission	118 (67.4)	24 (20.3)	38 (32.2)	40 (33.9)	16 (13.6)
Needed respiratory support in 24 hours	63 (36.0)	7 (11.1)	20 (31.8)	25 (39.7)	11 (17.5)
Needed vent in 24 hours	19 (10.9)	5 (26.3)	5 (26.3)	7 (36.8)	2 (10.5)
Admitted to ICU	25 (14.3)	4 (16.0)	9 (36.0)	9 (36.0)	3 (12.0)
Died in hospital	8 (4.6)	1 (12.5)	1 (12.5)	3 (37.5)	3 (37.5)

## Discussion

Analyzing the above data from 470 patients seen in the ER at Mercy Hospitals Toledo, subjects with hypertension and age > 50 years were found to be at the highest risk for hospital admission whereas predictors of increased mortality included increasing age >70 years, hypertension, diabetes, COPD, and CKD. However, there was no statistically significant difference in mortality between Whites and African Americans. Hypertension in itself was found to be the most significant factor in determining the need for hospital admission, respiratory support, ICU admission, and risk of death.

The first detailed Covid-19 series included 1099 hospital patients with confirmed Covid-19 out of China out of which mechanical ventilation was only required in 25 patients (2.3%) and 15 (1.4%) died [12]. However, later studies showed a much higher rate of hospital and ICU admission with one recent report from the Basque Country, Spain revealing 5775 (30%) of a total of 18,768 patients requiring hospital admission but only 448 (2.39%) with critical illness requiring mechanical ventilation or ICU support [13]. This is in contrast to our study in which a much higher patient population (14.7%) required ventilator support with a mortality of 10.9%. This might be because the majority of our patients had one or more co-morbidities (62.8%) with 226 patients (48.1%) having 2 or more chronic medical conditions. In contrast, the prevalence of co-morbidities in the first study from China was much less with only a quarter of them having one or more medical conditions. Furthermore, our study only included symptomatic patients seen in the ED whereas the study from Basque Country included electronic records from the general population cohort.

Our study demonstrated a strong association with hypertension, older age and hospital admission which is in concordance with previous studies. A significant increase in mortality among older people > 70 years was demonstrated in our cohort with an OR of 8.3. Also, people aged 50 or more were almost 4 times at higher risk of dying from the disease independently. This reveals that age in itself is an extremely important risk factor in determining outcomes from the disease, more so than hypertension alone. This has been replicated across various studies, an initial report in March 2020 by the CDC revealed that 67% of reported Covid-19 cases were aged 45 or older, with 80% of hospitalized patients in the same age group [14]. Similarly, in a modeling study based on the Chinese population, the hospitalization for Covid-19 patients increased with advancing age, with a 1% rate among the 20–29 group, 4% from 50–59 group, and 18% in people aged 80 or above [15]. Our study demonstrated a sequential increase in mortality with increasing age. This is also in concordance with literature, in an analysis from the United Kingdom, the risk of people dying from Covid-19 related complications was 20 times in the 80+ subset as compared to those aged 50–59 years [16].

Similarly, the risk of death in hypertensive patients was also almost 3.6 times that of the control group. These findings are similar to a recent pooled analysis of 13 studies with 2,893 patients with Covid-19 pneumonia which revealed a 2.5-fold increased risk of death in hypertensive patients [17].

Although there was a trend for increased mortality in obese patients (BMI 30 or more) with an Odds ratio of 1.2, we did not have enough power to achieve statistical significance. Studies have, however demonstrated a significant positive association with a higher BMI and severity of Covid-19 infection. A recent meta-analysis revealed that obese patients were 2 to 3 times more at risk of exacerbation of Covid-19 pneumonia [18,19]. However, the exact role of obesity as an independent factor is not clear. There is no standard definition as to what defines obese, with different studies having heterogeneous definitions. Also, the majority of these patients usually have multiple co-morbidities such as hypertension or DM which could increase the risk on their own and confound the results.

Our analysis also demonstrated a trend towards decreased mortality (40%) in women as compared to the men, however, results were not statistically significant. A literature review reveals studies showing an increased risk for acquiring Covid-19 infection, a higher severity of disease and mortality in men [20]. One study revealed that in similar age groups, men with covid-19 were more likely to have adverse outcomes as compared to women [21]. Unfortunately, an age-stratified analysis comparing men and women was not possible in our study due to paucity of data but our study did reveal a trend towards a higher risk of death in men. Similarly, the association of COPD and CKD with severe disease and mortality has also been replicated across major studies [22,23].

Diabetes Mellitus (DM) also remains a major risk factor for predicting mortality and morbidity in Covid-19 patients. Our study demonstrated a high prevalence of DM amongst total patients (40.4%), which is to our knowledge the highest prevalence amongst all reported studies so far, with an incidence ranging from 7.4%-20% previously [24–27]. It’s still unclear whether diabetics are more likely to get Covid-19 however, data is compelling that Diabetes is associated with higher adverse outcomes, ICU admission, and increased mortality in such patients [28]. Although the mechanism is not clearly understood, some studies show that the pro-inflammatory cascade might be exacerbated in diabetics leading to an inappropriate immune response and worse outcomes in such patients [29–31].

## Limitations

Our study had various strengths as well as some limitations. We were able to analyze the data from a relatively large database, which included a diverse subset of population from different races with a wide array of co-morbidities. We also had excellent follow up on most of the patients, with the use of state of the art Electronic Medical Record. Although the nature of our study is observational and a propensity-matched analysis was not possible, we did have significant power to arrive at potentially useful conclusions. One major limitation was that our population subset was from a single geographical region and although diverse, was treated under a single health care system. Thus, our data should be interpreted with caution when generalizing it to the general population as factors associated with poor health outcomes might differ in other places.

## Conclusion

Our study proves that Age and hypertension are associated with significant comorbidity and mortality in Covid-19 Positive patients. Furthermore, people who were older than 70, and had hypertension, diabetes, COPD, or CKD had higher odds of dying from the disease as compared to patients who hadn’t. Subjects with hypertension also had significantly greater odds of other adverse outcomes.

## Supporting information

S1 Checklist (DOCX)
